# Interação Letal entre Síndrome Hemofagocítica e Insuficiência Cardíaca Recentemente Desenvolvida

**DOI:** 10.36660/abc.20190642

**Published:** 2021-03-03

**Authors:** Devrim Bozkurt, Sukriye Miray Kilincer Bozgul, Omer Emgin, Osman Butun, Timur Kose, Evrim Simsek, Mine Hekimgil, Salih Kilic

**Affiliations:** 1 Ege University Faculty of Medicine Department of Internal Medicine Izmir Turquia Ege University Faculty of Medicine - Department of Internal Medicine, Intensive Care Unit Sectio, Izmir - Turquia; 2 Ege University Faculty of Medicine Department of Bioistatistics and Informatics Izmir Turquia Ege University Faculty of Medicine - Department of Bioistatistics and Informatics, Izmir - Turquia; 3 Ege University Faculty of Medicine Izmir Turquia Ege University Faculty of Medicine – Cardiology, Izmir - Turquia; 4 Ege University Faculty of Medicine Department of Pathology Izmir Turquia Ege University Faculty of Medicine - Department of Pathology, Izmir - Turquia; 5 Health Sciences University Adana Research and Training Hospital Department of Cardiology Adana Turquia Health Sciences University, Adana Research and Training Hospital - Department of Cardiology, Adana – Turquia

**Keywords:** Insuficiência Cardíaca, Linfo-Histiocitose Hemofagocítica, Inflamação, Mortalidade

## Abstract

**Fundamento:**

A síndrome hemofagocítica (SHF) é uma síndrome hiperinflamatória debilitante. O status da insuficiência cardíaca (IC) com fração de ejeção preservada (ICFEP) está intimamente relacionado ao aumento da inflamação sistêmica e intramiocárdica.

**Objetivos:**

este estudo pretende determinar os preditores de mortalidade e os parâmetros de monitoramento confiáveis nos casos de SHF que desenvolveram a ICFEP durante seu curso clínico.

**Métodos:**

Trinta e nove pacientes, diagnosticados com SHF de acordo com os critérios diagnósticos do estudo HLH 2004 com Hscore ≥169, e com aspiração ou biópsia de medula óssea comprovada, foram recrutados retrospectivamente. Foram investigados retrospectivamente os fatores de risco tradicionais, como proteína C reativa sérica, níveis de albumina e ferritina com contagens de linfócitos e plaquetas, e fatores não tradicionais, como relação neutrófilolinfócito (NLR), relação linfócito-monócito (MLR), volume plaquetário médio (MPV) e pró-peptídeo natriurético cerebral N-terminal (NTproBNP). Analisou-se a relação entre os valores laboratoriais alterados ao longo do tempo entre si e com a mortalidade. O nível de significância geral foi de 5%.

**Resultados:**

Foi demonstrado que a alteração temporal dos níveis de índice cardiotorácico (ICT), NTproBNP sérico, ferritina, PCR e albumina foram detectados como sendo preditores de mortalidade (p<0,05, para todos) em análise univariada. As contagens de linfócitos e plaquetas com valores de NLR e MPV também foram significativos (p<0,05). A relação entre NT-proBNP e o aumento dos marcadores inflamatórios sistêmicos também foi considerada significativa. Além de fatores de risco tradicionais, os níveis de ferritina sérica, e os níveis de NLR, MLR e MPV foram considerados significativamente correlacionados entre si.

**Conclusão:**

Acompanhado de parâmetros de monitoramento confiáveis, o diagnóstico rápido e o tratamento antiinflamatório agressivo com controle rígido de volume podem salvar vidas de pacientes com SHF que sofrem de complicações por ICFEP. O monitoramento rígido da inflamação pode prever o resultado do paciente que sofre de ICFEP.

## Introdução

A síndrome hemofagocítica (SHF) é um protótipo debilitante de hiperinflamação sistêmica grave. É uma síndrome clínica que frequentemente é letal como consequência da invasão de órgãos e tecidos por linfócitos T aumentados com excesso de citocina por inflamação contínua em indivíduos com distúrbio de eliminação de patógeno. O diagnóstico dos pacientes pode ser confundido com o de sepse devido a achados inespecíficos, tais como, febre, hepatoesplenomegalia, linfadenomegalia, enzimas hepáticas elevadas e citopenias. Os índices de mortalidade são altos devido tanto a isso quanto ao diagnóstico tardio.^1^ Nessa síndrome, que é caracterizada por tempestade de citocinas excessiva, muitos órgãos podem ser danificados irreversivelmente, e, na ausência de um tratamento anti-inflamatório e/ou anticitocinas, a morte é inevitável. O coração, inquestionavelmente afetado pelo aumento da inflamação, é o órgão mais proximamente associado ao sistema imune dentro de todos os sistemas de órgãos. A relação entre a supressão miocárdica e o aumento da inflamação é um fato conhecido. Outro protótipo de estudos de inflamação aumentada nos indivíduos com sepse demonstrou que a maioria dos pacientes têm mais manifestação clínica de insuficiência cardíaca (IC) com fração de ejeção preservada (ICFEP) nos estágios iniciais.

Como consequência disso, os sinais e sintomas de pacientes com SHF são muitos e podem levar à internação hospitalar com insuficiência cardíaca descompensada. A ICFEP, em aproximadamente metade dos pacientes com internação hospitalar devido à insuficiência cardíaca, é diagnosticada com base nos sinais e sintomas de insuficiência cardíaca, função sistólica ventricular esquerda normal ou apenas levemente anormal (fração de ejeção ventricular esquerda >50%), e evidências de disfunção diastólica. As pressões de enchimento ventricular elevadas são a anormalidade hemodinâmica proeminente em insuficiência cardíaca crônica e aguda. Muitos estudos anteriores demonstraram alto nível de status pró-inflamatório na circulação periférica e no coração de pacientes de IC. Além disso, esses estudos enfatizaram que a existência de um estado repetitivo e progressivo de ativação imunoinflamatória está fortemente associada à progressão da disfunção diastólica ventricular e da ICFEP.^2^^–^^9^

Se aceitamos a SHF como o protótipo de inflamação sistêmica grave, não é surpresa que a ICFEP se desenvolva no curso da SHF. Ainda temos poucas informações sobre o tratamento comprovadamente eficiente para pacientes de ICFEP além das medidas de suporte, incluindo modificação do estilo de vida, gestão da hipertensão, e controle metabólico de diabetes e obesidade. Recentemente, a estimulação da via da proteína quinase G parece abrir o horizonte para o futuro. Após esse entendimento, houve complicações de pacientes de SHF pela insuficiência cardíaca, este estudo desejou investigar a presença de possíveis parâmetros de inversão ou quaisquer preditores nesses pacientes que foram acompanhados no passado. Levantou-se a hipótese de que a inflamação é a principal fonte de depressão miocárdica e, se o estado hiperinflamatório for parado, a desintegração do miocárdio pode ser revertida.^9^ Nesse contexto, este estudo pretende determinar os preditores de mortalidade e os parâmetros de monitoramento confiáveis nos casos de SHF que desenvolveram a ICFEP durante seu curso clínico.

## Materiais e Métodos

### Pacientes

Dados de pacientes (n=63) que foram hospitalizados devido a SHF de acordo com os critérios diagnósticos do estudo HLH 2004 entre janeiro de 2012 e dezembro de 2018 foram coletados retrospectivamente.^10^^,^^11^ Pacientes com SHF com aspiração ou biópsia de medula óssea comprovada, com relatórios de ecocardiogramas detalhados, maiores de 18 anos, e hospitalizados por mais de 3 dias com score de hemofagocitose (Hscore)^6^ de 169 ou mais forma incluídos no estudo. O grupo portador de insuficiência cardíaca com fração de ejeção preservada (ICFEP) foi definido de acordo com as Diretrizes ESC de 2016.^10^ O histórico médico detalhado de cada paciente, incluindo os primeiros achados clínicos e laboratoriais referentes à SHF, foi registrado retrospectivamente. Por fim, trinta e nove pacientes, que têm resultados de análises hemobioquímicas e novos sintomas de insuficiência cardíaca, desenvolvidos durante a internação hospitalar, foram incluídos no estudo. Pacientes com internação hospitalar ≤ 3 dias, com histórico de malignidade ou quimioterapia prévios e sem dados suficientes, inclusive relatório de eletrocardiogramas e valores laboratoriais para o diagnóstico de SHF, foram excluídos do estudo.

### Fontes de dados

Dados de pacientes foram avaliados retrospectivamente por quatro médicos especialistas, incluindo cardiologista, hematologista, reumatologista e intensivista. As características clínicas, o histórico médico, e os parâmetros laboratoriais foram registrados dos registros dos pacientes e do sistema de dados digital do hospital retrospectivamente. O tempo (T-a-t, em dias) entre o primeiro sintoma ou dados laboratoriais que sugerem SHF e o início do tratamento efetivo também foi determinado. No momento do diagnóstico, o Hscore foi calculado de acordo com o relatório de Fardet et al.,^6^ Relatórios de hemogramas completos, incluindo a contagem de leucócitos polimorfonucleares (PMN), contagem de monócitos, contagem de linfócitos (L) contagem de plaquetas (PLT) e volume plaquetário médio (MPV) foram registrados. Todos os níveis de pró-peptídeo natriurético cerebral N-terminal (NTproBNP) durante a internação hospitalar foram registrados. A NLR foi obtida dividindo-se a contagem de neutrófilos circulantes absoluta pela contagem de linfócitos. A MLR foi obtida dividindose a contagem de monócitos pela contagem de linfócitos. Os relatórios de exames ecocardiográficos transtorácicos foram realizados por um cardiologista experiente conforme recomendado pela Sociedade Americana de Ecocardiografia (*American Society of Echocardiography*)^12^ e coletados retrospectivamente. Além disso, o índice cardiotorácico (ICT) para pacientes com insuficiência cardíaca também foi calculado de acordo com radiografias torácicas registradas anteriormente.^13^ O ICT foi calculado dividindo-se a largura horizontal máxima do coração pelo diâmetro interno da caixa torácica. A análise foi feita pela mesma equipe operacional utilizando-se software de computação para garantir a precisão da medida. Os dois valores laboratoriais dos pacientes no momento da internação hospitalar (linha de base, B) e no final da hospitalização (final, F), seja por óbito ou por alta, foram examinados. Alterações temporais de todos os valores durante a internação hospitalar foram representadas por ‘∆’.

Este estudo foi realizado de acordo com a Declaração de Helsinki e foi aprovado pelo comitê de ética médica do Hospital Universitário da Universidade Ege, em Izmir, na Turquia.

### Análise estatística

A compatibilidade das variáveis contínuas com a distribuição normal foi verificada separadamente em cada um dos grupos pelo teste de Shapiro-Wilk. As variáveis categóricas foram definidas como uma contagem resumida, em forma de porcentagem, enquanto as variáveis contínuas foram definidas como uma média resumida, faixa interquartil (FIQ). Optou-se por utilizar métodos não paramétricos, já que a maioria das variáveis numéricas não apresentam distribuição normal (teste U de Mann Whitney e análise de correlação de Spearman) Variáveis categóricas foram analisadas pelo teste Qui-quadrado, e as variáveis numéricas foram analisadas pelo teste U de Mann-Whitney. As diferenças entre os grupos de mortalidade e não mortalidade foram avaliadas pelo teste U de Mann Whitney. Foram realizadas análises de correlação de Spearman entre os parâmetros do grupo de mortalidade. O nível de significância geral é 5% O IBM Statistical Package for the Social Sciences (SPSS), Statistics for WindoWs, Version 25 (IBM Corp., Armonk, NY, USA), foi utilizado para a análise.

## Resultados

Um total de 39 pacientes (n=25, 64,1% mulheres), idade média 45,0 (22,0) anos foram incluídos no estudo. Entre eles, 10 (25,6%) pacientes evoluíram a óbito (grupo de mortalidade) e 29 pacientes receberam alta do hospital (grupo de sobrevivência). As características clínicas de linha de base e os parâmetros laboratoriais dos dois grupos foram resumidos na Tabela 1. Não houve diferenças significativas entre os grupos em termos de parâmetros laboratoriais. Não houve histórico médico de pacientes, exceto por dois pacientes hipertensos no grupo de sobrevivência. As etiologias subjacentes eram: doença infecciosa (n=10), doenças reumatológicas (n=20), malignidade (n=3), e origem idiopática (n=6). Quatro pacientes com etiologia de doença infecciosa, 2 pacientes com doença reumatológica, 3 com malignidade, e 1 com origem idiopática morreram. Durante o período de internação hospitalar, sete pacientes passaram por procedimento de hemodiálise, especialmente para atingir a ultrafiltragem adequada, e 25 pacientes receberam infusão de diurético de alça devido a hipervolemia.

**Tabela 1:** Comparação de características de linha de base e parâmetros laboratoriais

Variável	Grupo de mortalidade Média (FIQ) (n=10)	Grupo de sobrevivência Média (FIQ) (n=29)	p
Idade, anos	47,0 (29,0)	44,5 (22,0)	0,764
Sexo, feminino %	60 (n=6)	63,3 (19)	0,855
Pressão sanguínea sistólica	96,5 (27,0)	96,5 (21)	0,464
Pressão sanguínea sistólica	59,0 (17,0)	60,0 (15,0)	0,173
Hscore	223 (100)	229,0 (39,0)	0,868
Duração da internação hospitalar, dias	10,0 (6,0)	15,0 (15,0)	0,379
NT-proBNP (ng/L)	2390 (69930)	4000 (69960)	0,746
Ferritina (mcg/L)	4303 (169700)	21110 (98300)	0,216
Fibrinogênio (mg/mL)	257 (171)	318 (272)	0,289
Sedimentação (horas)	65,0 (46,0)	66,0 (41,0)	0,553
RNI	1,1 (1,9)	1,1 (1,2)	0,161
Albumina (mg/dL)	2,9 (3,0)	3,0 (2,4)	0,842
Globulina (mg/dL)	2,6 (0,8)	3,3 (1,0)	0,088
Ureia (mg/dL)	44,0 (122)	349,0 (268)	0,128
Creatinina (mg/dL)	0,73 (6,04)	0,8 (11,11)	0,202
LDH	583,0 (8840)	404,0 (5540)	0,197
PCR	6,32 (34,26)	8,4 (36,54)	0,406
Hemoglobina (g/dL)	9,7 (11,1)	8,7 (6,9)	0,204
Plaqueta × 10^3^	66 (442,0)	115,5 (749,0)	0,479
Neutrófilos	3500,0 (27200)	5800,0 (7800,0)	0,606

Foram realizados teste U de Mann Whitney para variáveis contínuas, e teste Qui-quadrado para variáveis categóricas. PCR: proteína C-reativa, FIQ: faixa interquartil, LDH: desidrogenase láctica, NTproBNP: Pró-peptídeo natriurético cerebral N-terminal.

**Figura 1:**
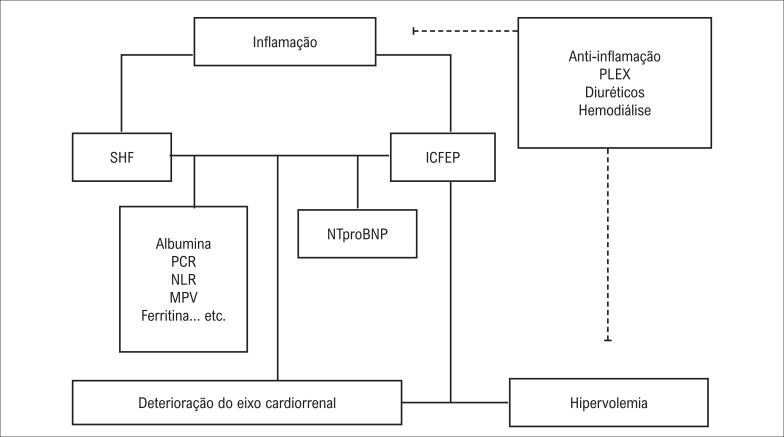
Cruzamento entre SHF e ICFEP. SHF, síndrome hemofagocítica. NTproBNP: Pró-peptídeo natriurético cerebral N-terminal; PCR: proteína C reativa; NLR: razão neutrófilo-linfócito; MPV: volume plaquetário médio; ICFEP: insuficiência cardíaca com fração de ejeção preservada; PLEX: plasmaferese.

Não houve diferença significativa no volume líquido (em litros) da ultrafiltragem entre o grupo da mortalidade e o grupo da sobrevivência (11,5 (3,8) e 10,5 (6,0) p=0,408, respectivamente). Os valores laboratoriais finais de pacientes vivos ou falecidos com toda a população do estudo foram apresentados na Tabela-2. O tempo médio decorrido entre os primeiros sintomas e o início de um tratamento anti-inflamatório adequado (T-a-t), ICT, NT-proBNP, PCR, ferritina, LDH, MLR e MPV final foram significativamente mais altos no grupo da mortalidade que no grupo da sobrevivência. Além disso, a albumina média, a contagem de linfócitos, e a contagem de plaquetas foram estatisticamente significativamente mais baixos no grupo de mortalidade que no grupo de sobrevivência. Durante a internação em CTI, a alteração temporal de ICT, e de níveis de NTproBNP sérico, ferritina, PCR e albumina, foram detectados como preditores de mortalidade (p<0,05, para todos). As contagens de linfócitos e plaquetas com valores de NLR e MPV também foram significativas (p<0,05 para todos) (Tabela 3). As alterações temporais do valor de MLR não atingiram a significância estatística (p=0,052). A análise de correlação de Spearman demonstrou correlações significativas entre os parâmetros de risco tradicionais e não tradicionais com alteração temporal (Tabela 4). Este estudo esquematizou a interação letal entre SHF e ICFEP via marcadores laboratoriais e o eixo cardiorrenal na figura 1.

**Tabela 2:** Valores laboratoriais finais de pacientes falecidos e vivos durante internação hospitalar

Variável		Grupo de mortalidade (n=10) Média (FIQ)	Grupo de sobrevivência (n=29) Média (FIQ)	p
T-a-t (em dias):		24,0 (60,0)	10,0 (28,8)	0,001
ICT (%)		56,0 (37,0)	50,0 (56,71)	0,026
NTproBNP (×10^3^ ng/L)		2,7 (70,0)	1,1 (25,7)	0,023
Albumina (mg/dL)		2,7 (2,5)	3,3 (2,1)	0,035
PCR (mg/dL)		7,9 (41,7)	1,4 (19,0)	0,005
Ferritina (×10^3^ mcg/L)		2,58 (120,0)	0,64 (1,53)	0,047
LDH (×10^3^ U/L)		0,56 (5,9)	0,2 (0,8)	<0,001
L (×103/µL)		0,77 (9,4)	1,5 (4,2)	0,010
PLT (×10^3^/µL)		47,0 (418,0)	188,5 (546,0)	<0,001
NLR		7,6 (52,2)	2,79 (14,4)	<0,001
MPV	Linha de base	11,7 (7,1)	11,7 (6,40)	0,035
	Final	11,8 (9,1)	10,5 (5,1)	0,027
MLR		1,0 (1,0)	0,5 (0,2)	<0,001

PCR: Proteína C reativa; ICT: Índice cardiotorácico; FIQ: faixa interquartil; NLR: Relação neutrófilo-linfócito; L: Contagem de linfócitos; LDH: Desidrogenase láctica; MPV: Volume plaquetário médio; MLR: Relação linfócito-monócito; NTproBNP: Pró-peptídeo natriurético cerebral N-terminal; PLT: Contagem de plaquetas; T-a-t (em dias): Tempo decorrido até o início do tratamento. Foi realizado o teste U de Mann Whitney para variáveis contínuas.

**Tabela 3:** Alteração temporal de preditores de mortalidade

Parâmetros	Grupo de sobrevivência (n=29)	Grupo de mortalidade (n=10)	p
	Média (FIQ)	Média (FIQ)	
Δ NTproBNP (×10^3^ng/L)	−4,67 (12,35)	28,26 (49,91)	0,007
Δ ICT (%)	−10,1 (4,00)	4,0 (10,00)	0,001
Δ Ferritina (×10^3^mcg/L)	−3,42 (18,08)	42,11 (179,83)	0,020
Δ LDH (×10^3^ U/L)	−0,31 (0,43)	−2,0 (3,68)	0,571
Δ PCR (mg/dL)	−6,8 (16,98)	0,0 (11,15)	0,001
Δ Albumina (g/dL)	0,40 (0,95)	−0,35 (0,75)	0,026
Δ L (×10^3^/µL)	0,53 (0,75)	−0,85 (660)	0,001
Δ PLT (×10^3^/µL)	68,05 (119,00)	−29,5 (56,25)	0,001
Δ NLR	−2,77 (8,37)	4,6 (12,09)	0,001
Δ MPV	−1,2 (1,92)	0,85 (1,8)	0,040
Δ MLR	−0,11 (0,57)	0,34 (1,61)	0,052

‘∆’ (Delta) significa a alteração em qualquer valor de parâmetro laboratorial durante a internação hospitalar. Ele é calculado como valor final, alta ou óbito, menos o valor de linha de base que é são os primeiros dados bioquímicos laboratoriais. PCR: Proteína C reativa; ICT: Índice cardiotorácico; FIQ: faixa interquartil; NLR: Relação neutrófilo-linfócito; L: Contagem de linfócitos; LDH: Desidrogenase láctica; MPV: Volume plaquetário médio; MLR: Relação linfócito-monócito; NTproBNP: Pró-peptídeo natriurético cerebral N-terminal; PLT: Contagem de plaquetas. Foi realizado o teste U de Mann Whitney para variáveis contínuas.

**Tabela 4:** Análise de correlação de Spearman entre parâmetros de risco

	CC (R^2^)	p		CC (R^2^)	p
Δ NTproBNP			Δ ICT		
Δ ICT	0,432	0,095	Δ NLR	0,461	0,031
Δ Ferritina	0,587	0,027	Δ MPV	0,561	0,004
Δ Albumina	−0,520	0,022	Δ MLR	0,404	0,041
Δ PCR	0,498	0,039	Δ PLT	−0,651	0,001
Δ PLT	−0,488	0,047	Δ PCR	0,411	0,041
Δ NLR	0,705	0,001	Δ NLR		
Δ MLR	0,478	0,038	Δ PCR	0,597	0,001
Δ Albumina			ΔFerritina	0,592	0,002
Δ NLR	−0,417	0,013	Δ PLT	−0,601	0,002
Δ MPV	−0,334	0,046	Δ L		
Δ Ferritina	−0,397	0,049	Δ Ferritina	−0,507	0,010
Δ PLT	0,341	0,039	Δ CRP	−0,531	0,001

Delta (‘∆’) reflete a alteração temporal de qualquer parâmetro durante a internação hospitalar. PCR: Proteína C reativa; ICT: Índice cardiotorácico; NLR: Relação neutrófilo-linfócito; L: Contagem de linfócitos; LDH: Desidrogenase láctica; MPV: Volume plaquetário médio; MLR: Relação linfócito-monócito; NTproBNP: Pró-peptídeo natriurético cerebral N-terminal; PLT: Contagem de plaquetas.

## Discussão

O presente estudo demonstrou que a alteração temporal de marcadores laboratoriais tradicionais (níveis de PCR sérico, albumina e ferritina com contagens de linfócitos e plaquetas), e não tradicionais (contagens de NLR, MPV, NTproBNP e ICT) de inflamação aumentada estão significativamente associados à mortalidade de pacientes com SFH durante o período de acompanhamento. A relação entre NT-proBNP e o aumento dos marcadores de inflamação sistêmica também foi considerada significativa.^14^ Além de fatores de risco tradicionais, as alterações temporais dos níveis de ferritina sérica, e dos níveis de NLR, MLR e MPV foram consideradas significativamente correlacionados entre si.^15^^–^^17^

O fato de que a contagem de plaquetas, que é o reagente positivo da fase aguda, pareça ser um reagente negativo da fase aguda neste estudo está relacionado à interrupção do consumo de plaquetas no caso de hemofagocitose possivelmente controlada. A relação entre a contagem de linfócitos e o bem-estar, que foi demonstrada em várias condições inflamatórias clínicas, também foi confirmada neste estudo.^18^^,^^19^ Esses são os primeiros parâmetros de triagem de pacientes com ICFEP. A principal causa de mortalidade entre os pacientes portadores de ICFEP se deve a motivos não cardíacos.^20^^,^^21^ As diretrizes não apresentam outro tratamento além do tratamento com diuréticos ou do tratamento com foco nas causas subjacentes até o momento.^9^^,^^21^ Recentemente, mostrou-se que o pró-peptídeo natriurético cerebral N-terminal (NT-proBNP) pode ter algumas vantagens.^10^^,^^22^ O NT-proBNP é uma ferramenta bem estabelecida para previsão da mortalidade de pacientes com insuficiência cardíaca com fração de ejeção preservada ou reduzida. Entretanto, há poucos dados sobre a relação entre esse parâmetro e a sobrevivência de pacientes durante a internação hospitalar. Neste estudo, mostrouse que a alteração temporal do NT-proBNP está intimamente associada à mortalidade de pacientes. O principal objetivo na ICFEP relacionada a SHF é impedir a presença de um status próinflamatório aumentado o mais breve possível. Do contrário, a morte é inevitável. O tempo decorrido desde o surgimento dos primeiros sintomas até o início do tratamento afeta diretamente a sobrevivência de pacientes, conforme demonstrado acima. Nos casos em que o tratamento rápido e eficiente foi iniciado, foi atingido um índice de mortalidade mais baixo, em comparação com os existentes na literatura.^22^ Na população do estudo, o índice de mortalidade geral é de 25,6%, e o índice de mortalidade causado por malignidade é de 19,4%. A deterioração do eixo cardiorrenal é outro cenário importante em pacientes de ICFEP.^23^ Devido ao aumento das pressões de enchimento ventricular, a maioria dos pacientes tem pressão venosa central e pressão intra-abdominais elevadas. O aumento da ativação do sistema renina-angiotensina e a redução do fluxo de plasma renal podem piorar as funções renais. Além disso, o carregamento inadequado de volumes leva ao aumento de pré-carregamento, com aumento dos sistemas de insuficiência e piora das funções renais. Também demonstrou-se que o equilíbrio de fluido positivo durante a disfunção diastólica está intimamente relacionado à mortalidade.^24^^,^^25^ Foi possível alcançar o controle rígido de volume, com procedimentos de infusão diurética contínua e ultrafiltragem. Da mesma forma, a melhoria nos testes de função renal apresentou benefícios para a sobrevivência. Isso reflete a recuperação da lesão renal aguda. Entretanto, nenhuma alteração atingiu significância estatística. O número pequeno de pacientes do estudo pode ser uma das causas. Com controle precoce e efetivo da inflamação e da hipervolemia, é certo que a sobrevivência vai aumentar nessa população.

## Limitações

Há algumas limitações neste estudo, uma das quais é o desenho retrospectivo. O risco de tendência do estudo não pode ser ignorado. Entretanto, os dados foram coletados por quatro pesquisadores que são especialistas em suas respectivas áreas. A etiologia de doença heterogênea pode ter um risco de mortalidade diferente. Entretanto, acredita-se que este estudo é muito importante para se determinar o risco de insuficiência cardíaca no curso da SHF, e para identificar os marcadores que afetarão a sobrevivência no monitoramento dos pacientes. Mesmo que não houvesse testes genéticos, medição de níveis de citocina, ou resultados de medições laboratoriais específicas, os pacientes foram meticulosamente selecionados, de acordo com os critérios do estudo HLH 2004 e com resultados do score de hemofagocitose.^11^^,^^26^ Deve-se observar que, devido às várias etiologias de SHF (incluindo lúpus eritematoso sistêmico, aparecimento da doença de Still do adulto, artrite reumatoide, doença mista do tecido conjuntivo, e causas infecciosas), o estudo enfrentou uma heterogeneidade inevitável.

## Conclusão

O presente estudo demonstrou que parâmetros sanguíneos simples e baratos, que podem ser obtidos com facilidade, podem ser um alerta precoce para os médicos durante o acompanhamento rotineiro de pacientes de SHF com complicação por ICFEP. Os parâmetros de monitoramento não tradicionais são valiosos nos casos de SHF com complicações por ICFEP, que é geralmente difícil de tratar e leva a altos índices de mortalidade. O estudo também propõe a interrupção da inflamação assim que possível para proteger o coração.
