# Supplementary material for: Interação Letal entre Síndrome Hemofagocítica e Insuficiência Cardíaca Recentemente Desenvolvida
Source: Arq Bras Cardiol. 2021 Mar 3;116(3):395–401. [Article in Portuguese] doi: 10.36660/abc.20190642 (PMC8159566; doi:10.36660/abc.20190642)
Supplement: Translation

# Translation: Mortal Interaction Between Hemophagocytic Syndrome and Newly Developed Heart Failure

**Authors:** Devrim Bozkurt, Sukriye Miray Kilincer Bozgul, Omer Emgin, Osman Butun, Timur Kose, Evrim Simsek, Mine Hekimgil, Salih Kilic

**Affiliations:** 1 Izmir Turkey Ege University Faculty of Medicine - Department of Internal Medicine, Intensive Care Unit Sectio, Izmir - Turkey; 2 Izmir Turkey Ege University Faculty of Medicine - Department of Bioistatistics and Informatics, Izmir - Turkey; 3 Izmir Turkey Ege University Faculty of Medicine – Cardiology, Izmir - Turkey; 4 Izmir Turkey Ege University Faculty of Medicine - Department of Pathology, Izmir - Turkey; 5 Adana Turkey Health Sciences University, Adana Research and Training Hospital - Department of Cardiology, Adana – Turkey

**Keywords:** Heart failure, Hemaphgocytic Lymphohistiocytosis, Inflammation, Mortality

## Abstract

**Background:**

Hemophagocytic syndrome (HPS) ia s devastating hyperinflammatory syndrome. Heart failure (HF) with preserved ejection fraction (HFpEF) status is closely correlated with increased inflammation, both systemic and intramyocardial.

**Objectives:**

This study sought to determine mortality predictors and reliable follow-up parameters in HPS that developed HFpEF during the clinical course.

**Method:**

Thirty-nine patients, diagnosed as HPS, according to HLH 2004 diagnostic criteria, with an HScore of ≥169 and proven bone marrow aspiration or biopsy, were recruited retrospectively. Both traditional, serum C-reactive protein, albumin and ferritin levels with lymphocyte, and platelet counts, as well as non-traditional risk factors, neutrophil-to-lymphocyte count (NLR), monocyte-to-lymphocyte count (MLR), mean platelet volume (MPV), and N-Terminal pro-brain natriuretic peptide (NTproBNP), were investigated retrospectively. The relationship between time-changed laboratory values both among themselves and with mortality. The overall significance level was set at 5%.

**Results:**

This study showed that temporal change of cardiothoracic ratio (CTR), serum NTproBNP, ferritin, CRP, and albumin levels were detected as mortality predictors (p<0.05, for all) in the univariate analysis. Lymphocyte and platelet counts with NLR and MPV values were also significant (p<0.05). The relationship between NT-proBNP and increased systemic inflammatory markers proved to be significant. In addition to traditional risk factors, serum ferritin levels, NLR, MLR, and MPV levels also proved to be significantly correlated with each other.

**Conclusion:**

Accompanied by reliable follow-up parameters, rapid diagnosis and aggressive anti-inflammatory treatment with tight volume control can be life-saving in HPS patients who suffer from HFpEF. Close monitoring of inflammation may predict the outcome of patients suffering from HFpEF.

## Introduction

Hemophagocytic syndrome (HPS) is a devastating prototype of severe systemic hyperinflammation. HPS is a clinical syndrome that is frequently lethal due to organ and tissue invasion of increased T lymphocytes with excess cytokine by continuous inflammation in individuals with pathogen clearance disorder. Patient diagnoses may be confused with sepsis due to its non-specific findings, including fever, hepatosplenomegaly, lymphadenomegaly, elevated liver enzymes, and cytopenias. Mortality rates are high both due to this and delayed diagnosis.^1^ In this syndrome, which is characterized by an excessive cytokine storm, many organs can be irreversibly damaged and death is inevitable in the absence of immediate anti-inflammatory and/or anti-cytokine treatment. The heart, undoubtedly affected by increased inflammation, is the organ most closely associated with the immune system within all organ systems. The relationship between myocardial suppression and increased inflammation is a well-known fact. Another prototype of increased inflammation studies in individuals with sepsis has shown that the majority of patients have a more heart failure (HF) with preserved ejection fraction (HFpEF) clinical picture in the early stages.

As a result, the signs and symptoms of patients with HPS is various and might refer to the hospital with decompensated heart failure. HFpEF, nearly half of the patients’ hospitalization due to heart failure, is diagnosed based on the signs and symptoms of heart failure, normal or only mild abnormal left ventricular systolic function (left ventricular ejection fraction >50%), and evidence of diastolic dysfunction. Elevated ventricular filling pressures are the prominent hemodynamic abnormality in both chronic and acute heart failure. Many previous studies have shown high levels of pro-inflammatory status in the peripheral circulation and heart of HF patients. Moreover, these studies emphasized that the existence of a repetitive and progressive state of immuno-inflammatory activation is strongly associated with the progression of ventricular diastolic dysfunction and HfpEF.^2^^–^^9^

If we accept HPS as the prototype of severe systemic inflammation, it is not surprising that HFpEF develops in the course of HPS. Information is still scarce in the literature on the most effective proven treatment for HFpEF patients other than supportive measures, including lifestyle modification, hypertension management, metabolic control for diabetes, and obesity. More recently, the protein kinase G pathway stimulation seems to open the horizon in the future. After it became clear that HPS patients were complicated by heart failure, it was important to investigate the presence of possible reversal parameters or any predictors in these patients that had been followed up on in the past. This study hypothesized that inflammation is the main source for myocardial depression, and the hyperinflammatory state were stopped, myocardial dysintegrity could be reversed.^9^ In this light, this study aimed to determine mortality predictors and reliable follow-up parameters in HPS that developed HFpEF during the clinical course.

## Material Method

### Patients

Data from patients (n=63) who were hospitalized due to the HPS, according to the HLH 2004 diagnostic criteria between January 2012 and December 2018, were collected retrospectively.^10^^,^^11^ HPS patients with proven bone marrow aspiration or biopsy, with detailed echocardiography reports, over 18 years of age, and hospitalized more than three days, with hemophagocytosis score (HScore)^6^ of 169 and above were included in the study. The HFpEF group was defined according to the 2016 ESC Guideline.^10^ Detailed medical history for each patient, including the first clinical and laboratory findings referring to HPS, were recorded retrospectively. Finally, 39 patients, who presented available blood biochemistry results and newly developed heart failure symptoms during hospitalization, were included in the study. Patient who had been hospitalized ≤3 days, presenting malignancy or a previous history of chemotherapy and no sufficent data,, including available electrocardiographic reports and HPS diagnostic laboratory values, were excluded from the study.

### Data sources

Patients’ data were evaluated retrospectively by four specialist phyicians, including an expert cardiologist, hematologist, rheumatologist, and intensive care physician. Clinical characteristics, medical history, and laboratory parameters were recorded from patients’ medical files and the hospital’s digital data system retrospectively. The time (T-to-t, in days) between the first symptom or laboratory data suggestive of HPS and the beginning of effective treatment was also determined. At the time of diagnosis, the HScore was calculated according to findings from Fardet et al.^6^ Complete blood count reports, including polymorphouse nucleated leucocyte count (PMNL), monocyte count, lymphocyte count (L), platelet count (PLT), and mean platelet volume (MPV) were recorded. All N terminal-pro brain natriuretic peptide (NTproBNP) levels during hospitalization were also recorded. NLR was obtained by dividing the venous absolute circulating neutrophil count by the lymphocyte count. MLR was obtained by dividing the monocyte count by the lymphocyte count. Transthoracic echocardiographic examination reports, performed by an experienced cardiologist, as recommended by the American Society of Echocardiography,^12^ were collected retrospectively. Likewise, the cardiothoracic ratio (CTR) for patients suffering from heart failure was also calculated according to previously recorded chest radiographies.^13^ The CTR was calculated by dividing the maximal horizontal width of the heart by the horizontal diameter of the inner borders of the rib cage. Analysis was performed by the same operational team, using computer software to ensure measurement accuracy. The two laboratory values of the patients at the time of hospitalization (baseline, B) and at the end of hospitalization (final, F), either dead or discharged, were examined. Temporal changes of all values during the hospital course were represented as ‘Δ’.

This study was conducted in accordance with the Declaration of Helsinki and was approved by the medical ethics committee at the Hospital of the Faculty of Medicine, Ege University, Izmir, Turkey.

### Statistical analysis

The compatibility of continuous variables to normal distribution was checked separately in each group by the Shapiro-Wilk test. Categorical variables were determined as a summarized count, in a percentage form, whereas continuous variables were determined as a summarized median, an interquartile range (IQR ). Non-parametric methods were preferred, as the majority of numerical variables failed to provide normal distribution (Mann Whitney U test and Spearman’s correlation analysis). Categorical variables were analyzed by the Chi-Square test and numerical variables were analyzed by the Mann-Whitney U Test. The differences between mortality and non-mortality groups were evaluated by the Mann Whitney U test. Spearman’s correlation analysis was performed between the mortality parameters. The overall significance level was set at 5%. The IBM Statistical Package for the Social Sciences (SPSS) Statistics for Windows, Version 25 (IBM Corp., Armonk, NY, USA), was used for analysis.

## Results

In total, 39 patients (n=25, 64.1% female), median of 45.0 (22.0) years of age, were included in this study. Of these, 10 (25.6%) patients died (mortality group), and 29 were discharged from the hospital (survival group). Baseline clinical characteristics and laboratory parameters of the two groups are summarized in Table 1. No significant difference was observed among the groups in terms of laboratory parameters. No medical histories of patients were found, except for two patients with hypertension in the survival group. The underlying etiologies were: infectious disease (n=10), rheumatological diseases (n=20), malignancy (n=3), and idiopathic origin (n=6). Four patients who presented infectious etiology, 2 with rheumatological disease, 3 with malignancy, and 1 of idiopathic origin died. During the hospitaliation period, seven patients underwent hemodialysis, primarily to achive adequate ultrafiltration, while 25 patients received loop-diuretic infusion due to the hypervolemia.

**Table 1:** Comparison of baseline characteristics and laboratory parameters

Variable	Mortality group Median (IQR) (n=10)	Survival group Median (IQR) (n=29)	p
Age, year	47.0 (29.0)	44.5 (22.0)	0.764
Sex, female %	60 (n=6)	63.3 (19)	0.855
Systolic blood pressure	96.5 (27.0)	96.5 (21)	0.464
Diastolic blood pressure	59.0 (17.0)	60.0 (15.0)	0.173
Hscore	223 (100)	229.0(39.0)	0.868
Hospitalization leght, day	10.0 (6.0)	15.0 (15.0)	0.379
NT-proBNP(ng/L)	2390 (69930)	4000(69960)	0.746
Ferritin (mcg/L)	4303 (169700)	21110 (98300)	0.216
Fibrinogen (mg/mL)	257 (171)	318 (272)	0.289
Sedimentation (hours)	65.0 (46.0)	66.0 (41.0)	0.553
INR	1.1 (1.9)	1.1 (1.2)	0.161
Albumine (mg/dL)	2.9 (3.0)	3.0 (2.4)	0.842
Globuline (mg/dL)	2.6 (0.8)	3.3 (1.0)	0.088
Urea (mg/dL)	44.0 (122)	349.0 (268)	0.128
Creatinine (mg/dL)	0.73 (6.04)	0.8 (11.11)	0.202
LDH	583.0 (8840)	404.0 (5540)	0.197
CRP	6.32 (34.26)	8.4 (36.54)	0.406
Hemoglobin (g/dL)	9.7 (11.1)	8.7 (6.9)	0.204
Platelet X 10^3^	66 (442.0)	115.5 (749.0)	0.479
Neutrophil	3500.0 (27200)	5800.0 (7800.0)	0.606

Mann Whitney U test for continuous variables, Chi-square test for categorical variables. CRP: c-reactive protein, IQR: interquartlie range, LDH: lactate dehydrogenase, NTproBNP: N-terminal pro-B-type natriuretic peptide.

There was no significant difference in the net ultrafiltration liters between the mortality group and the survival group (11.5 (3.8) and 10.5 (6.0), p=0.408, respectively). Final laboratory values of alive and deceased patients with the entire study population were presented in Table 2. The mean time spent from the first symptoms to the beginning of adequate anti-inflammatory treatment (T-to-t), CTR, NT-ProBNP, CRP, ferritin, LDH, MLR and final MPV were significantly higher in the mortality group than in the survival group. Moreover, the mean albumine, lymphocyte, and platelet counts were statistically much lower in the mortality group than in the survival group. During the ICU stay, the temporal change of CTR, serum NTproBNP, ferritin, CRP, and albumin levels were detected as mortality predictors (p<0.05, for all). Lymphocyte and platelet counts with NLR and MPV values were also significant (p<0.05, for all) (Table 3). Temporal changes in the MLR value failed to achieve the statitistical significance (p=0.052). Spearman’s correlation analysis showed the significant correlations between the traditional and non-traditional risk parameters with the temporal changes (Table-4). Trhis study schematized the lethal interaction between HPS and HFpEF via laboratory markers and the cardiorenal axis in Figure 1.

**Table 2:** Final laboratory values of deceased and living patients during hospital stay

Variable		Mortality group Median (IQR) (n=10)	Survival group Median (IQR) (n=29)	p
T-to-t (in days)		24.0 (60.0)	10.0 (28.8)	0.001
CTR (%)		56.0 (37.0)	50.0 (56.71)	0.026
NTproBNP (x10^3^ ng/L)		2.7 (70.0)	1.1 (25.7)	0.023
Albumin (g/dL)		2.7 (2.5)	3.3(2.1)	0.035
CRP (mg/dL)		7.9 (41.7)	1.4 (19.0)	0.005
Ferritin (x10^3^ mcg/L)		2.58 (120.0)	0.64 (1.53)	0.047
LDH (x10^3^ U/L)		0.56 (5.9)	0.2 (0.8)	<0.001
L (x10^3^/µL)		0.77 (9.4)	1.5 (4.2)	0.010
PLT (x10^3^/µL)		47.0 (418.0)	188.5 (546.0)	<0.001
NLR		7.6 (52.2)	2.79 (14.4)	<0.001
MPV	Baseline	11.7 (7.1)	11.7 (6.40)	0.035
	Final	11.8 (9.1)	10.5 (5.1)	0.027
MLR		1.0 (1.0)	0.5 (0.2)	<0.001

CRP: C-reactive protein; CTR: Cardiothoracic ratio; IQR: interquartlie range; NLR: Neutrophil-to-lymphocyte ratio; L: Lymphocyte count; LDH: Lactate dehydrogenase; MPV: Mean platelet volume; MLR: Monocyte-to-lymphocyte ratio; NTproBNP: N-terminal pro-B-type natriuretic peptide; PLT: Platelet count; T-to-t (in days): Time spent to begin treatment. For continuous variables, the Mann Whitney U test was performed.

**Table 3:** Temporal change of mortality predictors

Parameters	Survival group (n=29)	Mortality group (n=10)	p
	Median (IQR)	Median (IQR)	
ΔNTproBNP(x10^3^ng/L)	-4.67(12.35)	28.26(49.91)	0.007
Δ CTR (%)	-10.1(4.00)	4.0(10.00)	0.001
Δ Ferritin(x10^3^mcg/L)	-3.42(18.08)	42.11(179.83)	0.020
Δ LDH (x10^3^ U/L)	-0.31(0.43)	-2.0(3.68)	0.571
Δ CRP (mg/dL)	-6.8(16.98)	0.0(11.15)	0.001
Δ Albumin (g/dL)	0.40(0.95)	-0.35(0.75)	0.026
Δ L(x10^3^/µL)	0.53(0.75)	-0.85(660)	0.001
Δ PLT(x10^3^/µL)	68.05 (119.00)	-29.5 (56.25)	0.001
Δ NLR	-2.77(8.37)	4.6(12.09)	0.001
Δ MPV	-1.2(1.92)	0.85(1.8)	0.040
Δ MLR	-0.11(0.57)	0.34(1.61)	0.052

‘∆’ (Delta) refers to the change of any laboratory paramater value during hospital stay. It is calculated as the final value, either discharged or deceased, minus the baseline value, which is the first biochemical laboratory data. CRP: C-reactive protein; CTR: Cardiothoracic ratio; IQR: interquartlie range; NLR: Neutrophil-to-lymphocyte ratio; L: Lymphocyte count; LDH: Lactate dehydrogenase; MPV: Mean platelet volume; MLR: Monocyte-to-lymphocyte ratio; NTproBNP: N-terminal pro-B-type natriuretic peptide; PLT: Platelet count. For continuous variables, the Mann Whitney U test was performed.

**Table 4:** Spearman’s correlation analysis between risk parameters

	CC (R^2^)	p		CC (R^2^)	p
Δ NTproBNP			Δ CTR		
Δ CTR	0,432	0,095	Δ NLR	0,461	0,031
Δ Ferritin	0,587	0,027	Δ MPV	0,561	0,004
Δ Albumin	−0,520	0,022	Δ MLR	0,404	0,041
Δ CRP	0,498	0,039	Δ PLT	−0,651	0,001
Δ PLT	−0,488	0,047	Δ CRP	0,411	0,041
Δ NLR	0,705	0,001	Δ NLR		
Δ MLR	0,478	0,038	Δ CRP	0,597	0,001
Δ Albumin			Δ Ferritin	0,592	0,002
Δ NLR	−0,417	0,013	Δ PLT	−0,601	0,002
Δ MPV	−0,334	0,046	Δ L		
Δ Ferritin	−0,397	0,049	Δ Ferritin	−0,507	0,010
Δ PLT	0,341	0,039	Δ CRP	−0,531	0,001

Delta (‘Δ’) reflects the temporal change of any parameter during hospital stay. CRP: C-reactive protein; CTR: Cardiothoracic ratio; NLR: Neutrophil-to-lymphocyte ratio; L: Lymphocyte count; LDH: Lactate dehydrogenase; MPV: Mean platelet volume; MLR: Monocyte-to-lymphocyte ratio; NTproBNP: N-terminal pro-B-type natriuretic peptide; PLT: Platelet count.

**Figure 1:** A crisscross between HPS and HfpEF. HPS, hemophagocytic syndrome; NTproBNP: N-Terminal pro brain natriuretic peptide; CRP: C-reactive protein; NLR: Neutrophil-to-lymphocyte count; MPV: mean platelet volume; HfpEF: heart failure with preserved ejection fraction; PLEX: plasmapheresis.

## Discussion

The present study showed that the temporal changes of traditional (serum CRP, albumin, and ferritin levels with lymphocyte and platelet counts), and non-traditional (NLR, MPV, NTpBNP, and CTR counts) laboratory markers of increased inflammation are significantly associated with the mortality of patients with HSP during follow-up. The relationship between NT-proBNP and increased systemic inflammation markers proved to be significant.^14^ In addition to traditional risk factors, temporal changes of serum ferritin levels, NLR, MLR, and MPV levels proved to be significantly correlated with each other.^15^^–^^17^

The fact that the platelet count, which is a positive acute phase reactant, appears to be a negative acute phase reactant in this study, is related to the discontinuation of platelet consumption in the case of possibly controlled hemophagocytosis. The relationship between the lymphocyte count and well-being, which has been shown in many clinical inflammatory conditions, was also confirmed in this study.^18^^,^^19^ These are the first screening paramaters for patients with HFpEF. The main reason for mortality in patients with HFpEF is due to non-cardiac reasons.^20^^,^^21^ To date, the guidelines offer no treatment other than the treatment with diuretics or the treatment focusing on underlying causes.^9^^,^^21^ It was recently shown that the N-terminal pro-B-type natriuretic peptide (NT-proBNP) may have some advantages.^10^^,^^22^ NT-proBNP is a well-established tool to predict patient mortality in both heart failure with reduced or preserved ejection fraction. However, there is little data on the relationship between this parameter and patient survival during hospitalization. The present study showed that temporal changes of NT-proBNP are closely associated with patient mortality. The main goal of HPS-related HFpEF is to stop the presence of increased proinflammatory status as soon as possible; otherwise, death is inevitable. Time spent from the onset of first symptoms to the beginning of treatment directly affects patient survival, as shown above. Since rapid and effective treatment has been implemented, a lower mortality rate has been achieved, when compared to the literature.^22^ In our population, the overall mortality rate is 25.6% and the malignancy censored mortality rate is 19.4%. Cardiorenal axis deterioration is another important scene in HFpEF patients.^23^ Due to the high ventricular filling pressures, most patients present high central venous and intra abdominal pressure. Increased renin angiotensin system activation and reduced renal plasma flow may worsen renal functions. In addition, inappropriate volume loading leads to increased preloading, with an increase in failure symptoms, coupled with worsening renal functions. Positive fluid balance during diastolic dysfunction closely associated with mortality has been also shown.^24^^,^^25^ Strict volume control with continuous diuretic infusion and ultrafiltration procedures, as well as improvements in renal function tests, which provided survival benefits, were also achieved. This reflects the recovery of acute kidney injury. However, no changes reached statistical significance. The small number of study patients may play a role in this. With an early and effective control of inflammation and hypervolemia, it is certain that survival will increase in this population.

## Limitation

There are some limitations to this study, one of which is the retrospective design. The risk of bias in the study cannot be ignored. However, the data were collected by four researchers who are experts in their respective fields. The etiology of heterogeneous disease may have a different risk of mortality. However, we believe that this is a very important study to determine the risk of heart failure in the course of HPS and to identify the markers that will affect survival in patient follow-up. Even if there were no genetic tests, cytokine level measurements, and no specific laboratory measurement results, the patients were selected meticulously, according to the 2004 HLH study criteria and hemophagocytosis scoring results.^11^^,^^26^ It should be noted that, due to the different etiologies of HPS (including systemic lupus erythematosus, the onset of Adult Still’s disease, rheumatoid artritis, mixed connective tissue disease, and infectious causes), this study faced an inevitable heterogeneity.

## Conclusion

The present study illustrated that simple and cheap blood parameters, which can be easily obtained, may be an early warning for physicians during routine follow-up of HPS patients suffering from HFpEF. Our non-traditional follow-up parameters are valuable in HPS complicated with HFpEF, which is generally difficult to manage and leads to high mortality rates. This study also proposes the interruption of inflammation as soon as possible in order to protect the heart.
